# Molecular Dynamics Simulations of Atomic Diffusion during the Al–Cu Ultrasonic Welding Process

**DOI:** 10.3390/ma12142306

**Published:** 2019-07-19

**Authors:** Jingwei Yang, Jie Zhang, Jian Qiao

**Affiliations:** 1 School of Electromechanical Engineering, Foshan University, Foshan 528000, China; 2 State Key Laboratory of Tribology, Tsinghua University, Beijing 100084, China

**Keywords:** molecular dynamics, ultrasonic welding, diffusion

## Abstract

Ultrasonic welding (UW) is an important joining technique in the electrical industry. Molecular dynamic simulation has been shown to possess several advantages for revealing the evolution of the atomic-scale structure and the interpretation of diffusion mechanisms at the microscopic level. However, voids associated with the understanding of microstructure evolution in the weld zone and dynamic processes that occur during ultrasonically welded materials still exist, and no UW studies at the atomic scale have so far been reported. In this study, molecular dynamic simulations of UW between Al and Cu were performed to investigate the diffusion behaviors of Al and Cu atoms. The results confirmed the occurrence of asymmetrical diffusion at the Al/Cu interface during UW. Meanwhile, recovery was noticed in the disordered Al blocks at low temperature. The thickness of the diffusion layer increased with the welding time. For relatively long welding times (1 ns), the concentrations of Al and Cu revealed the appearance of phase transitions. In addition, the diffusion during UW was identified as a dynamic and unsteady process. The diffusion coefficient was much larger than that underwent during the steady diffusion process despite the low interfacial temperature (below 375 K), which was mainly attributed to shear plastic deformation at the interface.

## 1. Introduction

Ultrasonic welding (UW) is a solid-state joining process where two metal sheets are joined through high-frequency vibratory energy at a moderate clamping force. The progressive shear and plastic deformation caused by the elevated high frequency vibratory energy produces metallurgical coalescence between the different parts [1]. This makes UW insensitive to the thermal conductivity of the materials, making it particularly suitable for joining similar and dissimilar non-ferrous metals as well as alloys based on copper, aluminum, and magnesium, among other metals [2,3,4]. On the other hand, UW also has applications in other fields such as battery pack assembly, electrical wire connection, and light alloy structure welding.

With the rapid development of computer and molecular dynamics (MD), atomistic scale related research has attracted increasing attention. The MD method has been shown to possess several advantages for revealing the atomic-scale structure evolution and interpretation of diffusion mechanisms at the microscopic level. Hence, it has been widely used to study diffusion-related phenomena during welding processes. For instance, Zhu et al. [5] used MD to study the atomic behavior and inner atomic structure during the diffusion bonding of crystalline Al with metallic glass. Li et al. [6] investigated the effects of temperature and surface roughness on the diffusion welding between 304 SS and Ni at the atomic level using MD. Chen et al. [7] used the MD method to explore atom diffusion in Al/Cu explosive welding and the relationship between diffusion coefficients and collision velocity. Nikonov et al. [8] employed MD simulation to study the diffusion mechanisms induced by severe plastic deformation during friction stir welding. In a similar manner, Song et al. [9] investigated the MD of inter-diffusion at the Ti/Al joining surface during linear friction welding.

Numerous experimental and theoretical models dealing with UW of dissimilar metals such as Al/Cu [10,11], Al/Mg [12,13], and Mg/Ti [14] have so far been published. The weldability of various combined metals, the microstructural evolution of weld interfaces, and the optimized welding process parameters of interfacial metallurgical behaviors have so far been investigated. In addition, tremendous efforts have been made in thermo-mechanical analysis of the UW process through developed finite element models [15,16]. However, these experimental procedures are still limited by the spatial and temporal scales. Some experimental related phenomena like atom diffusion cannot directly be observed using conventional spatial and temporal scales. Therefore, voids associated with the understanding of microstructure evolution in the weld zone and dynamic processes occurring during ultrasonically welded materials still exist, and no UW studies at the atomic scale have so far been reported. 

In this study, a MD model of UW between Cu and Al was established. The atomic diffusion behaviors at the interface were explored and the dominant factors determining the diffusion process discussed. The results suggest that shear plastic deformation and elevated shear strain rate at the weld interface promote the diffusion process.

## 2. Simulation Methods 

**Figure 1:**
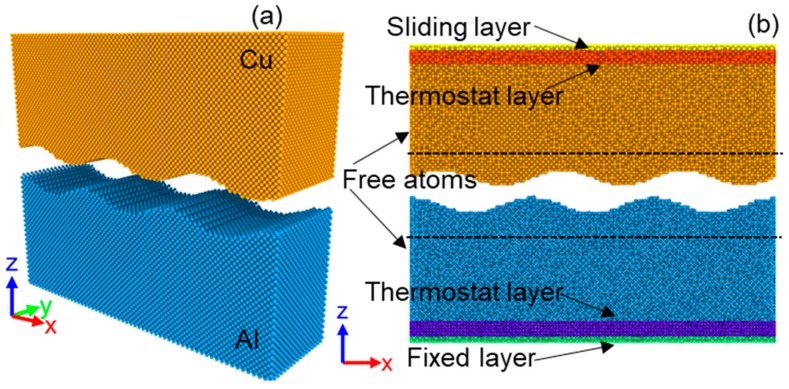
MD simulation model used for Al and Cu ultrasonic welding: (**a**) Initial configuration of sample; (**b**) Equilibrated configuration after relaxation under zero pressure at 300 K.

The LAMMPS package was employed to study the diffusion processes during UW [17]. The atomistic interactions were described in all calculations according to the embedded atom method developed by Cai and Ye [18]. Figure 1 illustrates the initial configuration of sample, consisting of a block of copper cell (up) and a block of aluminum cell (down). The dimension was set to 66L_Cu_(X) × 19L_Cu_(Y) × 28L_Cu_(Z) for Cu and 59L_Al_(X)× 17L_Al_(Y) × 25L_Al_(Z) for Al, where L_Cu_ = 0.362 nm and L_Al_ = 0.405 nm are the lattice constants of Cu and Al, respectively. The contact surfaces (x y) and the top surfaces of Cu and Al are both (0 0 1) planes. The practical surface between the slabs was not ideally smooth. Experiments have shown that the roughness of the contact surfaces has a significant impact on the bonding process. In our simulations, the surface was viewed as a sinusoidal distribution of asperities (Figure 1). The amplitude was set to 0.5 nm and the wavelength to 8 nm (1/3 of the block length). The system was initially equilibrated for 50 ps under zero pressure at 300 K. Thereafter, the equilibrated configuration of the sample is shown in Figure 1b, where the bottom two layers were set to be fixed and rigid. The top two layers were set to be rigid but movable along the x and z directions. The periodic boundary conditions were applied along the X and Y directions, while the boundary along the Z direction was free. Next to the bottom and top rigid layers, the Nosé-Hoover thermostat was employed to the thermostat zones, maintaining a system temperature of 300 K. All other atoms were free to move. The timestep was 1 fs. Before the welding, the system was allowed to relax for another 100 ps. The radial distribution function (RDF) and mean-squared displacement (MSD) values were calculated based on the atomistic information in the region between the dashed lines in Figure 1b.

**Figure 2:**
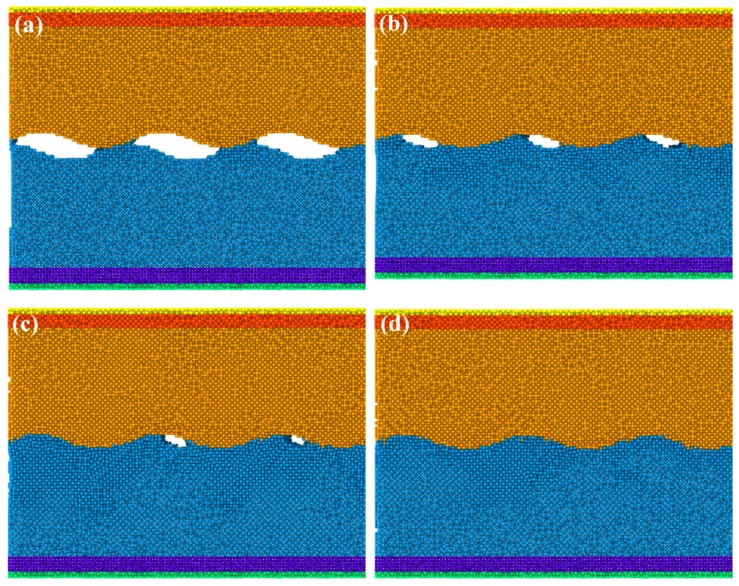
Configurations of the interfacial deformation process during the compression stage at: (**a**) 20 ps, (**b**) 40 ps, (**c**) 60 ps, and (**d**) 120 ps.

The UW process was divided into two stages: compression and vibration under constant pressure. Therefore, the Cu block at the beginning of the simulations was moved downward until reaching the contact surface of the Al block under constant pressure (Figure 2a). The compression stage lasted 100 ps (from 20 ps to 120 ps), as shown in Figure 2. The average applied pressure in the Z direction was 1 GPa. The crests at the copper surface subjected to pressure gradually pressed on the aluminum. The gaps between the grooves of the Al and Cu surfaces were filled by Al atoms from deformation, which were then transformed into pores, voids, and crests. The whole contact surface was finally obtained at 120 ps, indicating the immediate disappearance of interfacial gaps during the pressing stage.

The system was then relaxed at 300 K and under the pressure of 1 GPa for another 500 ps, and high-frequency motion was induced at the Cu slab in the X direction through the sliding layer. The motion was set according to the sinusoidal vibration, and the trajectory can be presented by Equation (1):

(1) S=Asin(2πft),

where *A* and *f* are the vibration amplitude and frequency. Since the spatial and temporal scales in MD are much smaller than the actual situation, in order to obtain the vibration effect, the selected vibration frequency should be much larger than the actual ultrasonic vibration frequency. Therefore, the vibration amplitude and frequency were set to 1 nm and 100 GHz, respectively [9].

## 3. Results and Discussion

### 3.1. Diffusion Behavior at the Interface

**Figure 3:**
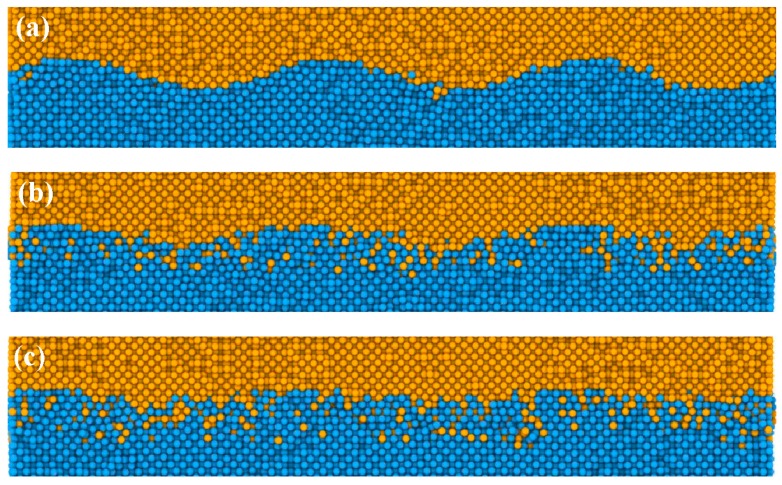

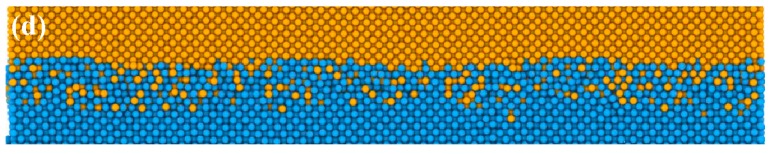
The cross-sectional views of the atomic configurations obtained at: (**a**) 0.1 ns, (**b**) 1 ns, (**c**) 3 ns, and (**d**) 5 ns. Only atoms near the interface are shown.

Figure 3 presents the evolution of interfacial diffusion behavior during the UW process for 5 ns. Snapshots of atomic configurations of the Al/Cu interface at 0.1, 1, 3, and 5 ns are gathered in Figure 3a–d. Some local atomic diffusion appeared along the interface after the occurrence of vibration, and the interface became gradually fuzzy despite the small number of atoms. A cross-sectional view containing the fuzzy interfaces at 0.1 ns is presented in Figure 3a. As time elapsed, more atoms diffused into each other to ultimately expand the Al/Cu interface into welding zones (Figure 3c,d). Two boundaries existed in the welding zone (WZ): one bounded by Al and the other by Cu. For convenient discussion, the boundaries were demoted as WZ-Al and WZ-Cu borders, respectively.

Obviously, only small amounts of aluminum diffused into the copper, but large numbers of copper atoms diffused into the aluminum block. Therefore, the migration velocity of the WZ-Al border was much faster than that of the WZ-Cu border, clearly suggesting the occurrence of asymmetric diffusion during welding. These data were consistent with previously reported experimental results for the UW of Al and Cu [11]. The reason for this has to do with the melting point of copper, which is higher than that of aluminum. Hence, the bonds in copper are much stronger than those in aluminum. Consequently, the face-centered cubic (fcc) structure of aluminum was easier to destroy while the original copper structure remained basically unchanged under this temperature. In addition, small atoms are more likely to diffuse into larger atoms [19], since the radius of the Cu atom is smaller than that of the Al atom, and asymmetrical diffusion occurs at the Al/Cu interface during UW.

**Figure 4:**
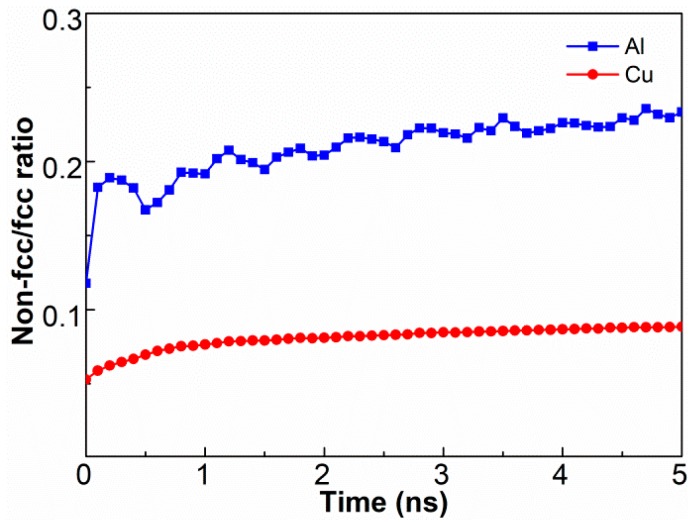
Quantity ratio between the non-fcc and fcc structures in the Al and Cu blocks as a function of welding time.

**Figure 5:**
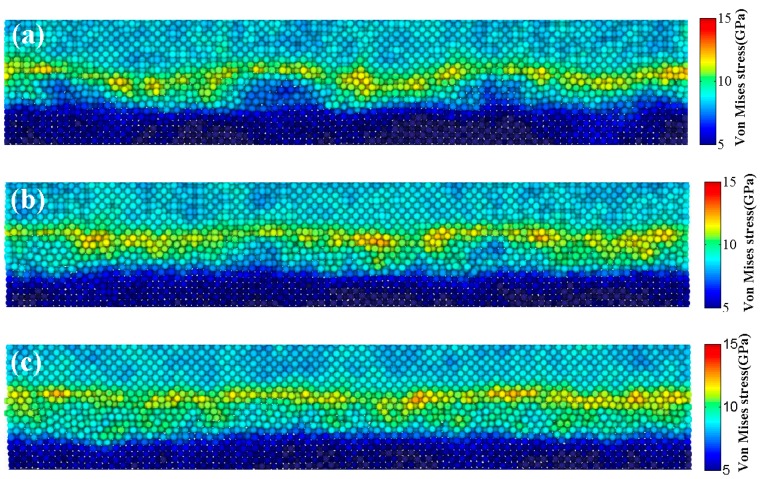
Stress distribution of the weld zone at: (**a**) 1 ns, (**b**) 3 ns, and (**c**) 5 ns.

Figure 4 displays the variation in atomic order and disorder for both aluminum and copper during the UW process. The quantity ratio between the non-fcc and fcc structures was employed to characterize the structural transformations. The number of non-fcc structures in the bulk materials increased with time to reach a maximum at 5 ns. Stress analyses were also conducted using MD and the results are depicted in Figure 5. Quite high-stress concentrates were noticed at the interface during welding, which were primarily attributed to plastic deformation induced by high-frequency sliding and pressure. Thus, the interface region was subjected to high and complex stress, leading to the deviation of aluminum and copper atoms from their original crystal structures and the transformation of large numbers of ordered structures into disordered arrangements. In addition, the ratio of non-fcc structures in Al was three-fold larger than that in Cu, confirming that a lattice structure of aluminum could be more easy to destroy than Cu, and is consistent with the reported literature [19]. At 0 ns, the deformation of crests at the surface during the pressing stage also promoted the structural transformation, clarifying why the ratio was not equal to 0. 

Another intriguing finding dealt with the visible fluctuations in the quantity ratio curve of Al resulting from plastic deformation induced by pressure and sliding at the interface region. During the welding process, the mechanical energy was transformed into heat and strain energy. This, in turn, increased both the interfacial temperature and stress concentration in the interface region, deviating large numbers of aluminum atoms from their equilibrium positions to form instantaneous vacancies. Meanwhile, part of the strain energy was released with the interface sliding, and the combination with heat promoted some deviated Al atoms to hop into the nearest neighbor vacancies. In other words, slight recovery occurred in the Al block during the UW process.

**Figure 6:**
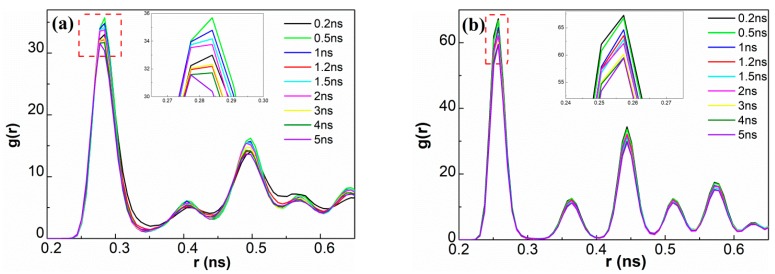
Variations in radial distribution functions of (**a**) Al–Al and (**b**) Cu–Cu at the weld interface during UW at different welding times. The insets show magnified views of the first peak.

The RDF g(r) would provide information related to local atomic density around a given atom. To further identify the changes in crystal structure, RDFs were carried out in the weld zone at different welding times and the data are compiled in Figure 6. For g(r) curves corresponding to the Cu–Cu and Al–Al pairs, high-intensity peaks were visible before the welding process, suggesting well-ordered structures. The peak intensity gradually decreased as welding time prolonged due to plastic deformation, indicating the formation of more defects and the destruction of long-range order in the weld region during the UW process. The inset of Figure 6a shows slight fluctuations in the magnitude of peak intensity for the Al–Al pair. However, unlike the Al–Al g(r) plots, the peak intensity in the Al–Al pair declined smoothly with time. These findings further confirmed the occurrence of recovery in distorted crystals at low temperature.

### 3.2. Concentration Profile and Diffusion Coefficients

**Figure 7:**
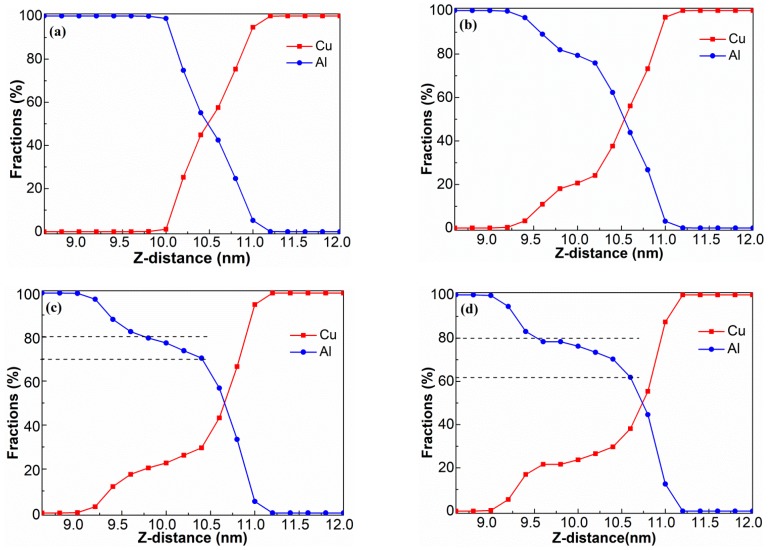
Variation in Al and Cu concentrations along the Z direction obtained at welding times of: (**a**) 0 ns, (**b**) 1 ns, (**c**) 3 ns, and (**d**) 5 ns.

To quantitatively characterize the two inter-diffuse processes, the specimens were cut into thin slices (0.2 nm thick) along the vertical direction of the interface, and then the Al and Cu concentrations were calculated (Figure 7a–d). The original interface showed a thickness scale around 1.2 nm at 0 ns (Figure 7a). The thickness of the diffusion layer gradually increased with time at 1 ns, 3 ns, and 5 ns (Figure 7c–d), consistent with the data in Figure 3. On the other hand, the ratios considerably varied in the diffusion layer, and the Al-rich transition layer looked much thicker than the Cu-rich layer. In Figure 7a,d, the DZ-Al border changed by about 1.3 nm (from 0.5 nm to 1.8 nm) while that of the DZ-Cu border varied by only 0.3 nm (from 0.5 nm to 0.8 nm). This reiterated the earlier discussion associated with the asymmetric diffusion during the diffusion process. 

Interestingly, concentration plateau profiles were observed after 1 ns. The width of the plateau became broader as the welding time increased. The corresponding concentrations of Al and Cu at the plateaus were determined as 60%–80% and 20%–40%, respectively (Figure 7b–d). The stoichiometric proportions of Al to Cu at the plateaus varied from 1.5 to 4, suggesting the appearance of phase transitions. Considering the Al–Cu binary phase diagram [20], the reaction–diffusion zone should likely consist of complex structures of Al and the disordered phase (Al_2_Cu), corroborating previously reported experimental results [6].

**Figure 8:**
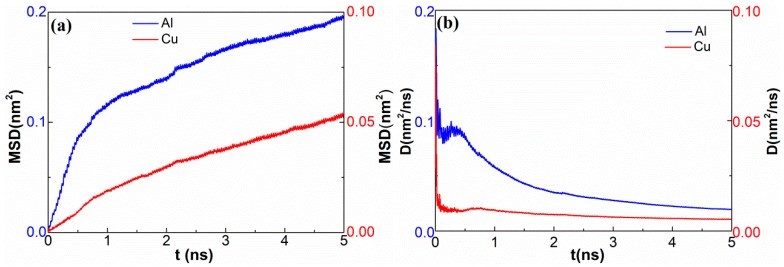
(**a**) MSD results of Al and Cu along the z-direction. (**b**) Results of running diffusion coefficients-half of slope of the MSD curves.

To gain a better understanding of the atom diffusion behaviors during UW, MSD values were calculated to figure out the motion state of the atoms in the deformed area near the interface. Figure 8a presents the MSD curves of Cu and Al in the z-direction. The MSD curves of Al and Cu were apparently divided into two stages. During the first stage, MSD showed a rapid linear increase with a larger rate. During the second stage, MSD slightly rose linearly, but kept increasing over time to reach 0.2 nm^2^ and 0.05 nm^2^ for Al and Cu at 5 ns, respectively. In addition to MSD, the running diffusion coefficients (RDC) of Al and Cu were also obtained by calculating half of the slope of the MSD curves along the z-direction. In Figure 8b, the slope of the MSD curve decreased gradually with time to reach a stable value, indicating dynamic and unsteady diffusion during the UW process. Moreover, the dynamic diffusion coefficient was about ten-fold larger than that of the reported steady diffusion welding at 800 K [21]. 

**Figure 9:**
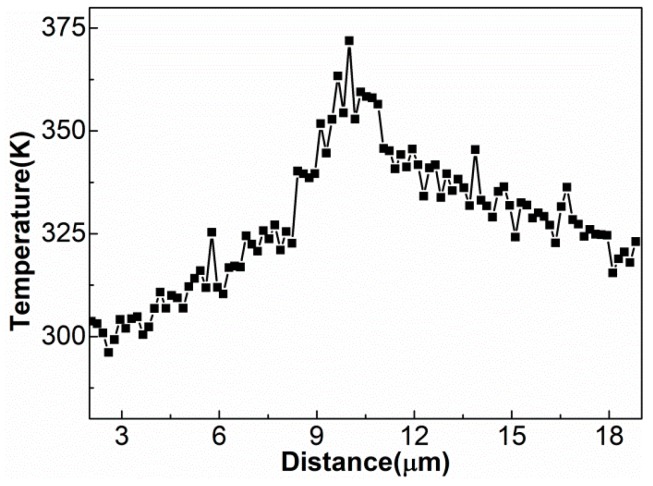
Temperature profile along the transverse cross-section calculated at 5 ns.

The diffusion between dissimilar metals often occurs at high temperatures since atoms require more energy to overcome the energy barrier for transition. However, the MD simulations showed that the increase in interfacial temperature during UW was less than 375 K (Figure 9). Hence, 375 K should not be high enough to promote a solid-state reaction. Obviously, the diffusion process was accelerated in UW, meaning that the reaction–diffusion during UW could not be attributed to heat but to stress and strain. On the other hand, severe plastic deformation of metals is usually accompanied by large shear deformation and high strain rate. The diffusion process should be accelerated more abruptly by shear deformation [22,23]. Since the crystal lattice could easily break during shear deformation, hence shear strain energy might help the atoms to overcome the energy barrier and lower the diffusion activation energy.

**Figure 10:**
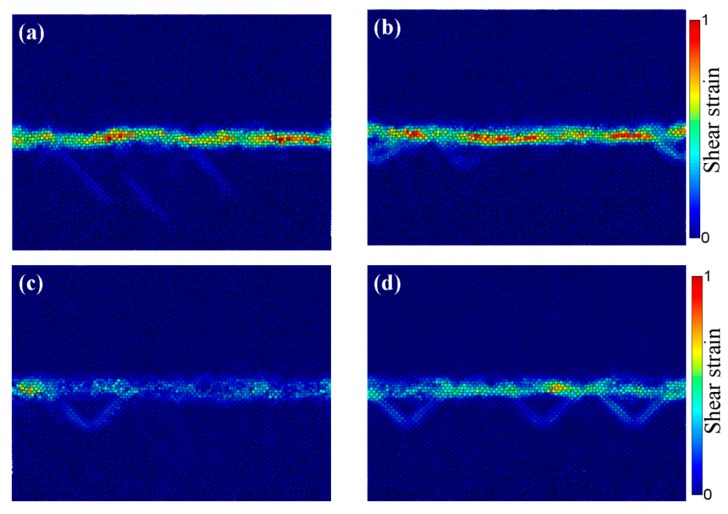
Shear strain distribution at different welding times: (**a**) 0.5 ns, (**b**) 1 ns, (**c**) 3 ns, and (**d**) 5ns.

The shear strain distributions at different welding times were extracted and the results are depicted in Figure 10. Apparently, a narrow shear plastic deformation band was observed at the weld interface. In addition, the shear strain greatly changed as the welding time rose, suggesting the deformation of the interfacial zone at high strain rates. As a result, large numbers of Cu and Al atoms migrated during UW from their original equilibrium positions to new positions on both sides of the adjacent interface. Meanwhile, elevated instantaneous vacancy concentrations formed during the welding process. The high strain rates in UW could increase the vacancy concentrations in Al up to 7 × 10^−2^ [24]. Thus, the vacancies could be considered as the best equilibrium positions for heterogeneous atoms for large-scale migration. Since the activation energy for high-density vacancy diffusion was much lower than that of lattice diffusion, the inter-diffusion between dissimilar atoms would occur when the vacancies in the Al side (or Cu side) of the Cu/Al interface became the new equilibrium position of Cu atoms (or Al atoms). The chemical reaction would occur between Cu and Al once the concentration of copper exceeded the solid solubility limit during Cu diffusion into Al, producing the intermetallic compounds. Therefore, the appearance of the plateau would indicate phase transitions induced by diffusion during UW.

## 4. Conclusions

The atom diffusion behaviors at the welding interface during Al–Cu ultrasonic welding were explored through molecular dynamic simulations. The following conclusions could be drawn:

(1) The difference in diffusion coefficients between Al and Cu induced a much faster migration velocity of the Al front than the Cu front, and asymmetric diffusion occurred at the interface during UW.
(2) During ultrasonic welding, the interface region was subjected to high and complex stress, transforming the perfect fcc structures into disordered arrangements with an increase in the number of non-fcc structures over time.
(3) The RDF peak intensity decreased as welding time increased, but the magnitudes of the peaks in the Al–Al pair fluctuated slightly, indicating the occurrence of low-temperature recovery during the welding process.
(4) The diffusion layer enhanced rapidly with welding time, and phase transitions occurred in the concentration profiles at a welding time of 1 ns.
(5) The diffusion during ultrasonic welding was identified as a dynamic and unsteady process. The diffusion coefficient appeared to be much larger than that obtained during steady diffusion, mainly attributed to shear plastic deformation and elevated shear strain rate at the weld interface.
